# Robotic and 3D laparoscopic radical nephroureterectomy with en bloc specimen excision (kidney, ureter, bladder cuff excision and extended lymphadenectomy) – Case report

**DOI:** 10.1016/j.ijscr.2022.106902

**Published:** 2022-03-01

**Authors:** Octavian Sabin Tataru, Eliza Cristina Bujoreanu, Bogdan Ovidiu Coste, Teodor Traian Maghiar, Bogdan Petrut

**Affiliations:** a I.O.S.U.D., George Emil Palade University of Medicine and Pharmacy, Sciences and Technology of Targu Mures, Department of Urology, Targu Mures, Romania; b Prof. Dr. I. Chiricuta" Institute of Oncology, Department of Urology, Cluj-Napoca, Romania; c Pelican “Hospital”- Medicover Group, Department of Urology, Oradea, Romania; d Iuliu Hatieganu" University of Medicine and Pharmacy, Department of Urology, Cluj-Napoca, Romania; e ESUT- EAU section of Uro-Technology Training Group, Romania

**Keywords:** Upper tract urothelial carcinoma, Radical robotic nephroureterectomy, Lymph node dissection, 3D laparoscopy, En bloc excision, Case report

## Abstract

**Introduction and importance:**

Upper tract urothelial carcinoma (UTUC) is a highly systemic aggressive disease with a tendency of rapid lymph node invasion and metastasis presenting poor oncologic outcomes. Ureteral localization of tumors leads to hydronephrosis and early invasion of the muscle wall, being categorized as high risk tumors.

**Case presentation:**

A 70 years old female was diagnosed with lower left ureteral urothelial tumor associated with hydronephrosis and paraaortic and iliac enlarged lymph nodes. The disease was stratified as high risk upper tract urothelial carcinoma. Treatment consisted in en bloc radical nephroureterectomy, bladder cuff excision and wide lymph node dissection using a combined robotic and 3D laparoscopic approach.

**Clinical discussion:**

Surgical challenges are surpassed by the use of minimal invasive approaches that offer precise dissection and tissue manipulation with a fast postoperative recovery and early adjuvant oncologic treatment. Comprehensive and complete lymph node dissection along with precise bladder cuff excision offers improved staging, possibly impacting disease prognosis.

**Conclusion:**

En bloc minimal invasive radical nephroureterectomy, bladder cuff excision and wide lymph node dissection offer improved surgery time and lymph node dissection, better management of distal ureteral and bladder cuff excision, watertight cystorrhaphy and optimal disease staging. The experience of the main surgeon with 3D laparoscopy was used in the hereby case to optimize operatory time for the renal step of the surgery. The gentle and precise movements of the Da Vinci robot allowed an accurate en bloc dissection (pN2, N4+/15) with implications in staging and possibly also in oncologic outcomes.

## Highlights

• Symptomatic patients presenting upper urinary tract urothelial carcinoma benefit from surgery, a minimal invasive approach being preferable.
• Nephroureterectomy with lymphnode metastasis poses surgical challenges in order to achieve negative resection margins and optimal lymphadenectomy.
• A combined 3D laparoscopic and Da Vinci X robotic surgical approach offered improved operatory time and better management of intra-pelvic disease. An optimal surgical strategy respects the surgical experience of the main surgeon combined with access to minimal invasive technology. The gentle and precise movements of the Da Vinci robot allowed an accurate dissection with en bloc specimen excision and optimal lymphadenectomy, presenting possible implications in staging and oncologic outcomes.

## 1 Introduction

Urothelial cancer (UC) is the fourth most common cancer [1]. The upper urinary tract (UTUCs) tumors account for 5–10% of UC [1]. Tumors in the kidney cavities (pyelocaliceal) are approximately 50% more common as ureteral tumors [2]. The first laparoscopic nephroureterectomy (LNU) was performed and published by Clayman, in 1991 [3]. Since then, advancements in technology placed LNU as a safe surgical treatment in UTUCs. Oncological outcomes after LNU or open radical nephroureterectomy (RNU) tend to be similar [2], with robotic radical nephroureterectomy (RRNU) offering advantages over LNU in terms of improved rates of lymph node dissection (LND) and short-term morbidity [4]. Veccia et al. [5], reported RRNU to be safe, providing the advantages of a minimally invasive approach without endangering oncologic outcomes, with further evidence from a systematic review suggesting that RRNU is being equivalent with LNU [6]. In a multicenter study, Roscigno et al. [7], found that pN+ is an independent predictor of cancer specific survival (CSS) (*p* < 0.001), therefore LND should improve the staging of the disease and establish the role of adjuvant chemotherapy in such patients. The hereby paper presents the case of a patient diagnosed with left ureteral distal urothelial cancer with latero-aortic and left iliac lymph node masses that underwent left en bloc radical Da Vinci X robotic nephroureterectomy with bladder cuff excision and extended lymph node dissection with the aid of 3D laparoscopy. The case was managed by a tumor board and no neoadjuvant treatment was given. The surgical technique and the analysis of *peri*/postoperative data are highlighted and oncologic management with 16 months follow-up data noted.

## 2 Presentation of case

A 70 years old female patient presented to our Department with gross haematuria, left nephralgia and lower left ureteral urothelial tumor associated with hydronephrosis and periureteral enlarged lymph nodes.

**Fig. 1:**
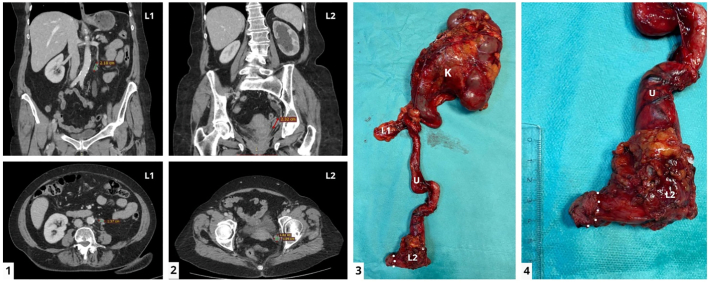
The Computed Tomography (CT) scan and en bloc excised specimen – macroscopic view. The CT (Computed Tomography) scan images (abdomino-pelvic, contrast enhanced-arterial phase) highlighting the latero-aortic lymph node mass (1.1) and left iliac lymph node mass in the vicinity of the ureteral tumor – on the distal ureter- with upstream ureterohydronephrosis (1.2). The images are presented in coronal (above) and axial (below) planes. The en bloc excised specimen (1.3) can be observed with a close-up on pelvic ureter (1.4). The kidney, ureter with perimeatic urinary bladder wall and attached lymph node masses (latero-aortic and left iliac) are marked. Symbols on images: K- left kidney, U - left ureter, L1 - latero-aortic lymph node mass, L2 – left iliac lymph node mass, DOTTED LINE - perimeatic left bladder wall.

Preoperative computed tomography (CT- abdomen, pelvis and thorax) confirmed the left distal ureteral tumor (20/17 mm) associated with grade III hydronephrosis (Fig. 1) and enlarged paraaortic and left external iliac lymph nodes. No distant metastases were noted. The disease is stratified as high risk UTUC, according to EAU Guidelines [2].

Laboratory examinations showed values within normal range: Hemoglobin level (11.1 g/dl), platelets level (381,000/μl), leukocytes level (8180/μl), Ca (9.2 mg/dl), K (3.81 mmol/l), Na (142 mmol/l), serum creatinine (1.05 mg/dl), serum urea (39.3 mg/dl). Urine analysis presented no pathological findings except hematuria and urine culture was negative. Karnofsky Performance status was 80 points, ECOG 1. The patient presented no medical/surgical/toxicological history. Analgesic medication was self-administered 3–4 times per week. The family history did not reveal any relevant genetic or psychosocial elements.

Informed consent has been obtained from the patient to use medical data and images. This work has been reported in line with the SCARE 2020 criteria [8].

### 2.1 Surgical technique

#### 2.1.1 Operating table position and port placement

**Fig. 2:**
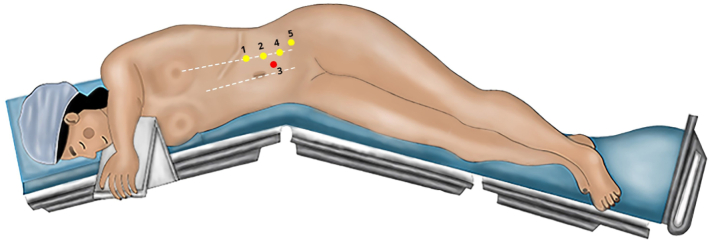
Patient positioning and trocar placement. Trocars placement for the 3D laparoscopic operatory steps: (1), (2) and (3). Robotic trocars placement for the Da Vinci X® operatory steps: (3), (4) and (5). (3) represents the optic trocar for both approaches.

The patient was placed in a flank position, with the table angled in the middle to expose the lumbar area. Trocar placement was performed for the initial step of 3D laparoscopic radical nephrectomy with cranial access ports and then adjusted for the Da Vinci® X robotic approach (Fig. 2).

#### 2.1.2 Radical robotic nephroureterectomy

##### 2.1.2.1 Radical 3D laparoscopic nephrectomy

After the descending colon was mobilized (Fig. 3.1) along the white line of Toldt, from the splenic flexure to the left iliac vessels, the retroperitoneum was accessed and the left lumbar ureter identified (Fig. 3.2). The dissection of the ureter advanced cranially with the en bloc excision of a latero- aortic lymph node mass (Fig. 3.3, 3.4, 3.5) and continued with the dissection of the renal pedicle. The renal artery followed by the renal vein were clipped and sectioned (Fig. 3.6, 3.7) and then the kidney was dissected from surrounding tissues (Fig. 3.8) and placed in an Endobag™ (Fig. 3.9).

**Fig. 3:**
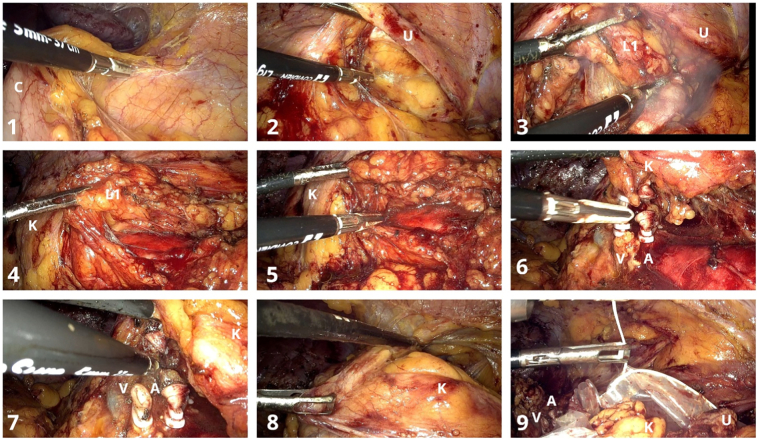
3D laparoscopic left radical nephrectomy and latero-aortic lymphadenectomy. Symbols on images: C – descending colon, K- left kidney, U - left ureter, L1 - latero-aortic lymph node mass, A - renal artery, V – renal vein.

#### 2.1.3 Robotic distal ureter dissection, lymph node dissection, bladder cuff excision and cystorrhaphy

The left ureter was dissected caudal (Fig. 4.1) as it crossed the iliac vessels and a lymph node was identified medial to the iliac vein (Fig. 4.1, 4.2, 4.3). The lymph node was dissected from the adjacent tissues but excised from the iliac fossa en bloc with the ureter (Fig. 4.4, 4.5, 4.6). The surgery continued with the perimeatic cystectomy (Fig. 4.7, 4.8) and the en bloc excision piece-nephroureterectomy with perimeatic cystectomy and attached lymph node masses (Fig. 4.9) was placed in 2 Endobags™ – one for the kidney and latero-aortic lymph node mass (initial step of the surgery – 3D laparoscopic) and one for the pelvic ureter with perimeatic cystectomy and iliac lymph node (second step of the surgery - Da Vinci X® robot).

**Fig. 4:**
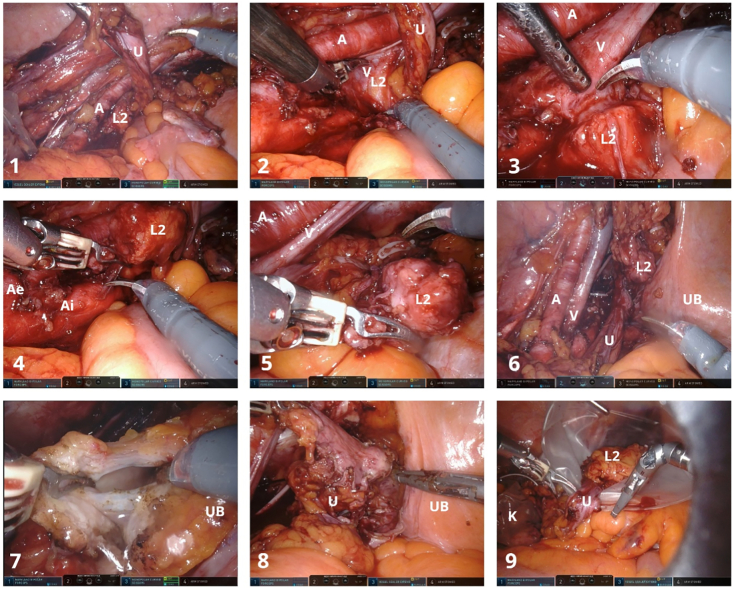
Da Vinci X® robotic left ureterectomy with perimeatic cystectomy and left iliac lymphadenectomy. Symbols on images: K- left kidney, U - left ureter, UB - urinary bladder, L2 – left iliac lymph node mass, A - external iliac artery, V – external iliac vein, Ae – external iliac artery, Ai – internal iliac artery.

The defect in the left wall of the bladder can be observed in Fig. 5.1 along with the inflated balloon of the transurethral indwelling catheter inside it. After a suprapubic catheter was placed, the cystorrhaphy was performed with a running suture (Fig. 5.2, 5.3, 5.4, 5.5, 5.6) using a Quill® thread. The en bloc specimen can be observed in Fig. 1.3, 1.4.

**Fig. 5:**
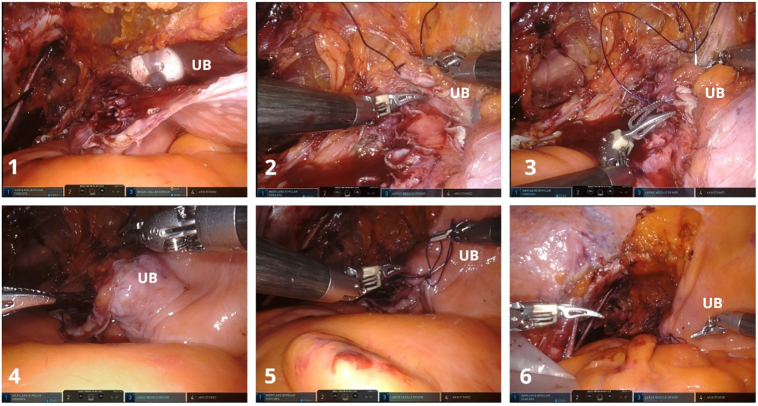
Da Vinci X® robotic cystorrhaphy. The cystorrhaphy was performed with a running suture using a Quill® thread. Symbols on images: UB - urinary bladder.

### 2.2 Results

Surgery time and perioperative results are presented in Table 1. The histopathologic examination revealed ureteral muscle invasive papillary urothelial carcinoma with lymphovascular invasion and negative surgical margins, G3 high grade tumor pT2N2(4+/15)V1Pn0R0, M0 status according to imagistic evaluation.

**Table 1:** Perioperative results and follow up data.

Results	
Total duration of surgery (minutes)	213
Operating room + patient preparation	35
Surgical Time (3D Laparoscopy + DaVinci® X robot)	160 (50 + 110)
Specimen extraction	6
Abdominal wall closure	12
Blood loss (ml)	100
Serum Creatinine levels (postoperative, mg/dl)	
3rd day	1.11
6 months	0.98
12 months	1.03
16 months	1.15
Clavien-Dindo (30 days) post operatory complications (grade)	I

The patient underwent adjuvant oncologic treatment as decided by a tumor board, with imaging (every 6 months), oncologic and urologic follow-up. The nephrologic exam showed no signs of renal insufficiency. Chemotherapy was administered 1 month after surgery as follows: 1 cycle of Gemcitabine and Cisplatin with intolerance to Cisplatin and continued with 4 cycles of Gemcitabine and Carboplatin. External radiotherapy followed after chemotherapy- 25 sessions, 45GY/28FR/1.8GY- paraaortic and left iliac vessels anatomic field.

At present date (16 months postoperative), the patient presents in good general status, with serum creatinine level in normal range and no imaging signs of disease recurrence or metastasis, but with moderate inferior left limb lymphedema (23% added circumferential difference). The patient reports a good life quality, using compression socks and lymphatic drainage massages weekly.

## 3 Discussion

One of the first described retroperitoneal RRNU was published in 2006 by Rose et al. [9], performed on two patients without conversion. In order to reduce surgery time and the morbidity after prolonged anesthesia a combined (3D laparoscopic and robotic) approach was preferred for the present case. Experiences of different techniques of distal ureter resection with bladder cuff excision [10] were reviewed in emerging studies that compared outcomes of laparoscopic vs. RRNU [6], [11], [12], [13]. Literature reviews on how to assess the clinical impact of lymphadenectomy [14], to compare different robotic platforms during RRNU [15] and different laparoscopic and robotic approaches with the open approach [16], [17] were published. Robotic LND is suited for dissection of large number of lymph nodes with less morbidity and improvements for disease staging [4]. Different techniques were described for patient positioning (45/60 degree flank position), with a tilted Trendelenburg position [18] bringing the advantage of a better kidney exposure that facilitates renal hilum, upper kidney pole access as well as ureteral dissection. Access ports were placed in a linear configuration to facilitate access for both the upper pole of the kidney and the urinary bladder and for pelvic LND to achieve accurate stratification of the disease, similar to what Taylor et al. [18], previously described. The distal management and excision of the bladder cuff is performed by the Da Vinci® systems (X, SI, XI) with improved dissection of the distal ureter and bladder cuff excision and have advantages because it forgoes cystoscopy and repositioning, and facilitates LND and cystotomy closure [10], [19]. This patient positioning on the operating table offered the space needed to quickly dock the robotic platform, therefore without losing time to readjust the surgical field.

Lymphadenectomy is advisable in patients with muscle-invasive UTUCs because the 5 year overall survival and cancer specific mortality are comparable between patients with N1 and N0 muscle-invasive UTUCs [14]. In the hereby case, 4 out of the 15 excised lymph nodes presented malignancy, establishing pT2pN2 staging. The number of excised lymph nodes shows a high quality excision of the lymph nodes, helped by the precision of the Da Vinci robot. In a retrospective analysis of 7278 patients with UTUC treated with RNU, Zhai et al. [20], found that a higher overall survival (OS) and CSS was associated with LND in patients with T3-T4 tumors (*p* < 0,05), but not in pT1 and pT2 disease (*p* > 0,05). A newer systematic review looked into the potential benefit of lymph node dissection and compared CLND, incomplete and no LND. CLND is as an independent prognostic factor for improved survival, but did not show significant survival differences for tumors located in the ureter. Reviews suggest that RRNU is similar for perioperative and oncological performance to other surgical techniques (LNU, open nephroureterectomy), but it may offer a lower overall complication rate as well as postoperative mortality, such as the results in our case with grade 1 postoperative (30 days) Clavien-Dindo complications [6]. Template for CLND for lower ureter tumors as described by Campi et al. [21], involves on the left side the obturatory, external, internal and common iliac lymph nodes and the para-aortic LND being a controversial topic. Our extended para-aortic LND offers better stratification for our patient. RRNU seems to be safe and offers the advantages of a minimally invasive technique respecting oncological principles [5]. It is well known that there is no consensus regarding the treatment approach for UTUC patients with nodal involvement, but in selected cases such as symptomatic patients, EAU guidelines recommend surgery as a palliative treatment [2]. Nonetheless, Covid-19 is putting its toll on cancer patients and their need for treatment, therefore UTUCs is considered a high risk disease and a high priority for patients to have access to treatment without any delay [22].

## 4 Conclusions

This case represents a successful management by a tumor board of a patient with a high grade ureteral muscle invasive papillary urothelial carcinoma with lymphovascular invasion pT2N2(4+/15)M0V1Pn0R0 that received adjuvant treatment (chemotherapy and radiotheraphy) after minimal invasive en bloc excision surgery.

RRNU can offer very good perioperative results, improved surgery time, and improved LND, better management of distal ureteral and bladder cuff excision, better watertight cystorrhaphy. Da Vinci X® may offer better access at the edges of the operatory field; either we are talking the bladder or the superior renal pole. Da Vinci X® may need trocar port translocation with one more trocar in long shaped patients. Access with only three ports (camera and two instruments) may offer easier access at the edges of the operatory field. But in this situation an assistant with laparoscopic skills may constitute a good advantage. The en bloc resection of kidney, ureter and lymph nodes helps the surgeon keeping track in removing all the lymphatic tissue in the designated areas, keeping the connection of lymph nodes with the ureter and for better stratification.

The experience of the leading surgeon with 3D laparoscopy was used to optimize operatory time for the renal step of the surgery, using the large jaws of the bipolar laparoscopic device. The precision offered by the Da Vinci X robot allowed an accurate en bloc dissection and intra-pelvic disease management, possibly influencing oncologic outcomes.

## Consent

Written informed consent was obtained from the patient for publication of this case report and accompanying images. A copy of the written consent is available for review by the Editor-in-Chief of this journal on request.

## Sources of funding

This research did not receive any specific grant from funding agencies in the public, commercial, or not-for-profit sectors.

## Ethical approval

This work does not require a deliberation by the ethics committee.

## Registration of research studies

Not applicable.

## Guarantor

Bogdan Ovidiu Coste accepts full responsibility for the work and had controlled the decision to publish.

## Provenance and peer review

Not commissioned, externally peer-reviewed.

## CRediT authorship contribution statement

**Tataru Octavian Sabin**: Conceptualization, Data curation, Formal analysis, Funding acquisition, Investigation, Methodology, Project administration, Resources, Software, Supervision, Validation, Visualization, Writing - original draft, Writing - review & editing, final approval of the version to be submitted.

**Bujoreanu Eliza Cristina**: Conceptualization, Data curation, Formal analysis, Funding acquisition, Investigation, Methodology, Project administration, Resources, Software, Supervision, Validation, Visualization, Writing - original draft, Writing - review & editing, final approval of the version to be submitted.

**Coste Bogdan Ovidiu**: Conceptualization, Data curation, Formal analysis, Funding acquisition, Investigation, Methodology, Resources, Supervision, Validation, Visualization, Writing - review & editing, final approval of the version to be submitted.

**Maghiar Teodor Traian**: Conceptualization, Data curation, Formal analysis, Funding acquisition, Investigation, Methodology, Project administration, Resources, Software, Supervision, Validation, Visualization, Writing - review & editing, final approval of the version to be submitted.

**Bogdan Petrut**: Leading surgeon, Conceptualization, Data curation, Formal analysis, Funding acquisition, Investigation, Methodology, Project administration, Resources, Software, Supervision, Validation, Visualization, Writing - review & editing, final approval of the version to be submitted.

## Declaration of competing interest

None. All authors report no conflict of interests or financial ties.
